# Supplementary figures and images for: The D-dimer level predicts the postoperative prognosis in patients with non-small cell lung cancer
Source: PLoS One. 2019 Dec 26;14(12):e0222050. doi: 10.1371/journal.pone.0222050 (PMC6932866; doi:10.1371/journal.pone.0222050)

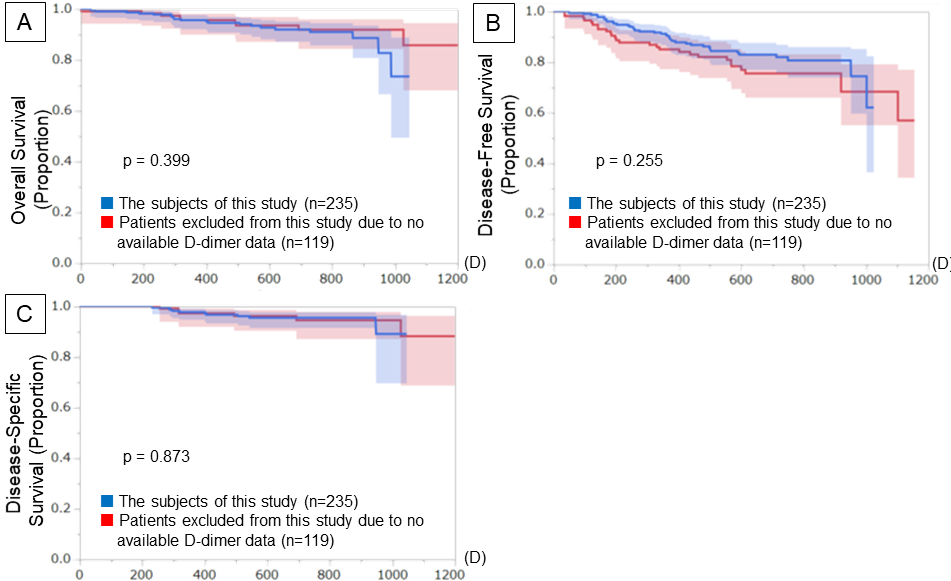

Supplement: S1 Fig — For the 235 subjects of this study and 119 excluded patients due to no available D-dimer data, Kaplan-Meier curves of postoperative overall survival (A), disease free survival (B) and disease specific survival (C) were compared. (TIF) [file pone.0222050.s001.tif]
