# Supplementary material for: The D-dimer level predicts the postoperative prognosis in patients with non-small cell lung cancer
Source: PLoS One. 2019 Dec 26;14(12):e0222050. doi: 10.1371/journal.pone.0222050 (PMC6932866; doi:10.1371/journal.pone.0222050)
Supplement: S1 Table — (DOCX) [file pone.0222050.s002.docx]

**S1 Table. Clinicopathologic profiles of the subjects of this study and patients excluded from the study**

|  |  | **Subjects of this study** | **Excluded patients*** | **P value** |
| --- | --- | --- | --- | --- |
|  |  | **n=235** | **n=119** |  |
| Age, years |  | 67.9±9.1 | 71.4±8.4 | **<0.001** |
| Gender |  |  |  | 0.730 |
|  | Male | 146 | 72 |  |
|  | Female | 89 | 48 |  |
| Tumor size, mm | | 26.6±17.3 | 24.3±15.8 | 0.203 |
| CT appearance | |  |  | 0.720 |
|  | Pure solid | 164 | 88 |  |
|  | Partly solid GGN | 13 | 7 |  |
|  | Pure GGN | 58 | 25 |  |
| Histology |  |  |  | 0.970 |
|  | Adenocarcinoma | 69 | 35 |  |
|  | Other | 166 | 85 |  |
| Micropapillary pattern | |  |  | 0.161 |
|  | (+) | 16 | 4 |  |
|  | (-) | 219 | 116 |  |
| p-Stage |  |  |  | 0.374 |
|  | I | 172 | 93 |  |
|  | II, III | 63 | 27 |  |
| p-pl |  |  |  | 0.725 |
|  | pl0 | 184 | 83 |  |
|  | pl1 | 28 | 17 |  |
|  | pl2 | 9 | 6 |  |
| p-pm |  |  |  | 0.220 |
|  | pm0 | 232 | 112 |  |
|  | pm1 | 1 | 2 |  |
|  | pm2 | 2 | 0 |  |
| p-ly |  |  |  | 0.433 |
|  | ly0 | 208 | 104 |  |
|  | ly1 | 27 | 10 |  |
| p-v |  |  |  | 0.947 |
|  | v0 | 176 | 85 |  |
|  | v1 | 59 | 29 |  |

GGN: ground-glass attenuation-dominant nodule, CT: computed tomography

*The patients were excluded from this study due to a lack of available D-dimer data.
