# Supplementary material for: The D-dimer level predicts the postoperative prognosis in patients with non-small cell lung cancer
Source: PLoS One. 2019 Dec 26;14(12):e0222050. doi: 10.1371/journal.pone.0222050 (PMC6932866; doi:10.1371/journal.pone.0222050)
Supplement: S2 Table — (DOCX) [file pone.0222050.s003.docx]

**S2 Table. Uni- and multivariate analyses for the OS in patients with a pure solid appearance**

|  |  | **Univariate analysis** | |  | **Multivariate analysis** | |
| --- | --- | --- | --- | --- | --- | --- |
|  |  | **Hazard ratio (95%CI)** | **P value** |  | **Hazard ratio (95%CI)** | **P value** |
| Age (years) | |  |  |  |  |  |
|  | <70 | - |  |  |  |  |
|  | ≥70 | 1.51 (0.62-3.78) | 0.359 |  |  |  |
| Gender | |  |  |  |  |  |
|  | Male | 1.97 (0.72-6.95) | 0.200 |  |  |  |
|  | Female | - |  |  |  |  |
| cN |  |  |  |  |  |  |
|  | 0 | - |  |  | - |  |
|  | 1 | 1.04 (0.06-5.17) | 0.970 |  | 1.28 (0.07-7.02) | 0.824 |
|  | 2 | 3.18 (0.50-11.30) | 0.183 |  | 3.88 (0.55-16.40) | 0.149 |
|  | 3 | - |  |  | - |  |
| cT |  |  |  |  |  |  |
|  | 1 | - |  |  | - |  |
|  | 2 | 0.46 (0.10-1.58) | 0.229 |  | 0.29 (0.16-1.12) | 0.075 |
|  | 3 | 3.55 (0.54-13.81) | 0.159 |  | 1.86 (0.28-7.60) | 0.446 |
|  | 4 | 8.22 (2.46-24.74) | **0.001** |  | 5.50 (1.42-18.09) | **0.011** |
| D-dimer (µg/mL) | |  |  |  |  |  |
|  | ≥1 | 5.54 (2.24-13.97) | **< 0.001** |  | 4.30 (1.67-11.56) | **0.003** |
|  | < 1 | - |  |  | - |  |

GGN: ground-glass attenuation-dominant nodule, CI: confidence interval, OS: overall survival
