# Supplementary material for: The D-dimer level predicts the postoperative prognosis in patients with non-small cell lung cancer
Source: PLoS One. 2019 Dec 26;14(12):e0222050. doi: 10.1371/journal.pone.0222050 (PMC6932866; doi:10.1371/journal.pone.0222050)
Supplement: Attachment — Submitted filename: Response to Reviewers190811.docx [file pone.0222050.s005.docx]

Dear editor

We wish to express our appreciation to the Reviewers for their insightful comments, which have helped us to significantly improve the paper.

We have added the following sentence about information about the patient records.

“All patients’ data were fully anonymized before we accessed them and the ethics committee of Chiba University waived the requirement for informed consent.”, to Page 8, Line 99

Response to Reviewers

Reviewer #1: The authors investigated whether D-dimer was a prognostic impactor for patients with non-small cell lung cancers. Although not that novel, the study was reliable and the analysis was appropriate.

1. What's the normal range of D-dimer in the institution?

Answer: The normal range of D-dimer was less than 1.0µg/ml. We have added the following sentence.

“a normal D-dimer (less than 1.0 µg/mL) group”, to Page 7, Line 74

2. Why ten patients received induction therapy?

Answer: Ten patients had mediastinal lymph node metastasis which was diagnosed as N2, stage IIIA lung cancer. The multidisciplinary team conference which was consisted of thoracic surgeons, respirologists, medical oncologists, and radiologists decided to perform multidisciplinary treatment including induction chemo-radiotherapy following surgery for these patients.

3. Since the small number of patients in each cN group, I wonder if it would be integrated into with or without lymph node metastasis.

Answer: Thank you very much for your suggestion. We have changed the table 1.

4. As for HDD group, any further examination or intervention has been used?

Answer: almost all patients in this study, both for HDD group and NDD group, underwent contrasted CT scan in order to exclude pulmonary artery embolism prior to thoracotomy.

5. Did surgical procedure (Lobectomy vs sublobar resection) impact the prognosis?

Answer: Thank you very much for your suggestion. There was no prognostic difference between the patients who underwent lobectomy or more and the patients with sublobar resection.

Reviewer #2: This is an interesting original work, and the only necessary thing to do is to have more appropriate references, shaping a bit different discussion, and thus making more profound and comprehensive explanations for your findings. Just to mention, vascular invasion should be in this context adequately discussed, as well as the tendency of more distant metastasis in the HDD group than in the NDD group although not significant difference in the type of recurrence, as well as NSCLC with a solid tumor appearance on CT in the context of the poorer prognosis. More recent references should be used - there are several large scaled prospective studies that found that the association of high D-dimer levels with poor prognosis was independent of VTE in hematologic malignancies and solid tumors, which raised the question that D-dimer might be able to affect prognosis through a VTE independent pathway, thus pointing that D-dimer may play an important role in the tumorigenesis.

Some suggested references, among necessary to add and comment:

-Lyman GH, Khorana AA. Cancer, clots and consensus: new understanding of an old problem. J Clin Oncol. 2009; 27:4821-4826.

-Zhao J, Zhao M, Jin B, et al. Tumor response and survival in patients with advanced non-small-cell lung cancer: the predictive value of chemotherapy-induced changes in fibrinogen. BMC Cancer 2012; 12:330.

-Zhu L, Liu B, Zhao Y, Liu L, Yang C, Yang Y, Zhong H. High levels of D-dimer correlated with disease status and poor prognosis of inoperable metastatic colorectal cancer patients treated with bevacizumab. J Cancer Res Ther. 2014; 10:246-251.

-Chen Y, Yu H, Wu C, Li J, Jiao S, Hu Y, Tao H, Wu B, Li A. Prognostic value of plasma D-dimer levels in patients with small-cell lung cancer. Biomed Pharmacother. 2016; 81:210-217.

Answer: Thank you very much for your very important comments and suggestions. As the reviewer suggested, recent publications suggested the correlation between D-dimer level and poor prognosis. We have added the suggested reference.

We have added the following sentences.

“Several prospective studies of other organs have shown that the association of high coagulation parameters with a poor prognosis was independent of venous thromboembolism [19] [20]. The D-dimer level might thus affect the prognosis solely through an important role in tumorigenesis separately from the venous thromboembolism pathway.”, to Page 21, Line 210

“Several reports have shown that tumor-mediated coagulation activation is associated with tumor growth, angiogenesis promotion and metastasis [21]. Platelets are reported to increase the metastatic success via multiple mechanisms, including direct shielding of tumor cells and protection of tumor cells from cytokines [22] [23]. The fibrinolytic system has been reported to promote tumor growth through several different mechanisms, including angiogenesis, suppressing apoptosis, proliferation of tumor cells and degradation of the extracellular matrix [24]. These previous findings may explain why the HDD group had more distant metastasis in the present study than NDD group did.”, to Page 21, Line 215

“Some studies have found that thrombin increased the invasiveness of cancer [25] [26], although no report has mentioned the coagulation system and vessel invasion. Although increased D-dimer levels might be a result of vessel injury due to tumor invasion, the association between the D-dimer levels and vessel invasion is unclear and more detailed studies are required”, to Page 22, Line 225

As the reviewer suggested, we reanalyze the recurrences, dividing the recurrences into local and distance recurrences. We have also added the following sentences, and modified the table 3.

“The HDD group had more distant recurrences than did the NDD group (p=0.045) (Table 3). In contrast, there was no significant difference in the local recurrence between the 2 groups (p=0.218).”, to Page 15, line 136
